# Investigating the Relationship between Ambulatory and Hospital Patient Experience Scores in a Neurosurgery Practice

**DOI:** 10.3390/healthcare9091153

**Published:** 2021-09-03

**Authors:** Richard Meyrat, Elaina Vivian, Jimmy Shah, Archana Sridhar, Bonnie Blake Hurst, Chris Shoup, Randall B. Graham, Stephen Katzen, Bartley Mitchell, Michael Oh, Nimesh H. Patel

**Affiliations:** 1 Methodist Moody Brain and Spine Institute, Dallas, TX 75203, USA; ArchanaSridhar@mhd.com (A.S.); RandallGraham@mhd.com (R.B.G.); StephenKatzen@mhd.com (S.K.); BartleyMitchell@mhd.com (B.M.); MichaelOh@mhd.com (M.O.); NimeshPatel@mhd.com (N.H.P.); 2 Methodist Dallas Medical Center, Methodist Digestive Institute, Dallas, TX 75203, USA; ElainaVivian@mhd.com (E.V.); JimmyShah@mhd.com (J.S.); 3 Clinical Effectiveness & Patient Safety, Methodist Health System, Dallas, TX 75203, USA; BonnieHurst@mhd.com; 4 Executive Office, Methodist Health System, Dallas, TX 75203, USA; ChrisShoup@mhd.com

**Keywords:** Press Ganey, patient satisfaction, neurosurgery practice, quality improvement

## Abstract

Patient experience is critically important on both clinical and business levels to healthcare organizations, medical groups, and physician practices. We sought to understand whether a relationship exists between patient satisfaction scores in different settings for medical providers who practice in multiple settings (such as in the ambulatory setting and the hospital) within a system. Press Ganey (PG) ambulatory and hospital-based patient satisfaction surveys of a neurosurgery practice were retrospectively compared. Questions and sections related to the care provider, likelihood to recommend, and overall experience were examined. The ambulatory dataset included 2270 surveys, and the hospital dataset included 376. Correlation analysis of hospital survey patients who also completed an ambulatory survey (*N* = 120) was conducted, and weak, yet statistically significant, negative correlations between hospital “Likelihood to Recommend” and ambulatory “Care Provider Overall” (r = −0.20421, *p* = 0.0279), “Likelihood to Recommend” (r = −0.19622, *p* = 0.0356), and “Survey Overall” (r = −0.28482, *p* = 0.0019) were found. Our analyses found weak, yet significant, negative correlations between ambulatory and hospital PG scores. This could suggest that patient perception established in ambulatory and clinic settings could translate to a patient’s perception of their hospital experience and subsequent satisfaction scores.

## 1. Introduction

Patient experience has affected the healthcare industry in pivotal ways over the last decade. It is at the top of the systemic clinical and business priority lists of healthcare organizations, medical groups, and physician practices across the country. Patient experience is also an extremely valuable consideration for consumers, and therefore an important outcome to consider. Jha et al. identified at least six different domains that factor into patient experience as a whole: personalization, admission and discharge process, patient safety, clinical effectiveness, patient engagement, and satisfaction [1].

From the business perspective, patient experience affects the bottom line, either positively or negatively, by impacting organizations and providers’ reputation among consumers and their eligibility to receive funding from Medicare [2]. Consumers are increasingly able to have a choice in their healthcare options, which they can navigate by investigating quality and cost factors online [3]. Companies like Healthgrades, which provides a large amassment of information on physicians, hospitals and health care providers to the general public, make consumer shopping comparisons possible by providing decision-making data [4]. Additionally, the public can review a hospitals’ current Hospital Consumer Assessment of Healthcare Providers and Systems (HCAHPS) scores on the Medicare website [5].

From the clinical perspective, the patient experience is related to several clinical processes and outcomes, and therefore should be a focus of improvement efforts. For example, patient experience has been positively correlated to processes of care for both prevention and disease management [6], and patient communication with providers was correlated to adherence to medical advice and treatment plans [7,8,9,10]. Patients that reported better care experiences often had better health outcomes [11,12,13,14].

The patient–provider relationship and communication influence patient satisfaction [15,16,17]. Chipidiza et al. defined four elements of the patient–provider relationship (i.e., trust, knowledge, regard, and loyalty), and showed that all four elements affect both patient outcomes and patient satisfaction [18].

A focus on patient experience benefits all involved. As a result, healthcare organizations, medical groups, and physician practices are looking to allocate resources towards the most meaningful strategies for quality improvement. While investigating new strategies to improve the patent experience domain specific to patient satisfaction, a question emerged that was unanswered in the currently available literature: For medical providers who have direct interactions with patients in multiple settings (e.g., the ambulatory setting AND the hospital), is there a relationship between patient satisfaction scores in the different settings? This was our primary objective, and we believe if such a relationship exists, it could infer that the patient–provider relationship and perception of care established in the ambulatory setting could translate to a patient’s perception of their hospital experience and subsequent satisfaction scores. Additionally, with so many factors going into how patients perceive and then rate their care experiences, an additional question for medical providers practicing in multiple settings would be whether their patient satisfaction scores are comparable between these settings? This was our secondary objective.

This paper investigates these research questions and objectives by describing a five-year retrospective study comparing Press Ganey (PG) ambulatory and hospital-based patient satisfaction scores of a neurosurgery practice.

## 2. Materials and Methods

### 2.1. Study Setting

Methodist Moody Brain and Spine Institute (MBSI) is an independent neurosurgical practice at not-for-profit Methodist Health System (MHS) in North Texas and Dallas, Texas. The MBSI provides treatments for back pain and neck pain, brain and spinal tumors, neurovascular/stroke conditions, spinal disorders, spinal cord injuries, and other neurological conditions.

### 2.2. Study Design and Participants

**Figure 1:**
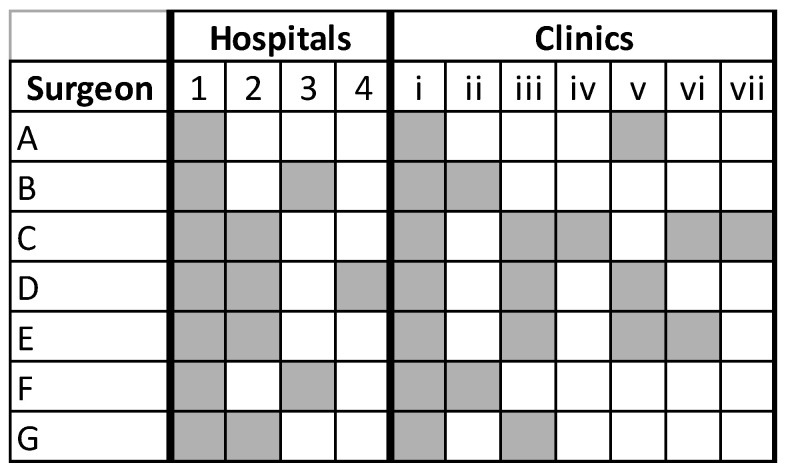
Neurosurgeon practice matrix.

A retrospective study was conducted of all MBSI’s neurosurgeons’ ambulatory and hospital-based PG patient satisfaction scores between January 2016 and December 2020. During this period, MBSI had seven neurosurgeons practicing throughout the MHS network of 7 ambulatory and 4 hospital facilities, as outlined in Figure 1. Hospital surveys were only pulled for patients where the attending physician listed was one of the 7 neurosurgeons. Institutional review board approval was obtained (Methodist Health System Institutional Review Board, Dallas TX); patient consent was not required.

All patients of the 7 MBSI neurosurgeons completing an ambulatory and/or hospital PG survey between January 2016 and December 2020 were included. Hospital surveys were only pulled for patients where the attending physician listed was one of the 7 neurosurgeons.

### 2.3. Study Outcomes

Results from PG ambulatory practice and hospital PG surveys were used to assess patient satisfaction, particularly focusing on questions and sections related to the care provider, likelihood to recommend, and overall experience. The primary outcome is the correlation coefficient between ambulatory practice and hospital PG questions. The secondary outcome were the mean scores of the ambulatory practice and hospital PG surveys.

Scores were tallied for the selected PG questions, PG survey sections, and the survey overall for each physician and for the entire neurosurgeon group.

Patients answered PG questions on a 5-point Likert scale, where 1 = very poor, 2 = poor, 3 = fair, 4 = good, and 5 = very good. These were then converted to a 0 to 100-point scale, where 1 = 0, 2 = 25, 3 = 50, 4 = 75, and 5 = 100, and the mean scores were calculated.

Selected PG sections and questions from the ambulatory survey included the following: (i) Care provider overall. The care provider domain questions included explanations the care provider gave you about your problem or condition; concern the care provider showed for your questions or worries, care provider’s efforts to include you in decisions about your care, likelihood of recommending this care provider to others, and care provider’s discussion of any proposed treatment (options, risks, benefits, etc.). (ii) Likelihood to Recommend care provider to others (single question). (iii) Survey overall. The mean survey score was calculated of all PG ambulatory survey domains which included: Access; Moving through Your Visit; Nurse/Assistant; Care Provider; and Overall Assessment.

Selected questions from the PG hospital survey included the following: (i) Doctors overall. The section score of care provider domains including the following: Time physician spent with you; Physician’s concern for your questions and worries; and How well physician kept you informed; (ii) Likelihood of recommending hospital to others (single question); (iii) Survey overall. The mean survey score of all PG hospital survey sections which included: Personal Issue Overall; Nurses Overall; Overall Assessment Overall; Doctors Overall; and Room Overall.

Ambulatory surveys were sent to patients 7 days after their visit. Due to the longitudinal nature of the study period, some patients completed multiple ambulatory surveys. Hospital surveys were sent 7 days after discharge. However, the hospital surveys were mailed surveys, so it could take a few additional days for patients to receive and return those surveys.

### 2.4. Statistical Analysis

Analyses were conducted using SAS v. 9.4 (SAS Institute Inc., Cary, NC, USA). Descriptive statistics are reported as mean (standard deviation [SD]) [range] for all continuous variables and as absolute (*n*) and relative frequencies (%) for categorical variables. Continuous variables were evaluated for normality using the graphical normal probability plot (i.e., QQ plot). Simple and multiple linear regression were used to determine any relationships between survey participants’ demographics and PG questions. One-way analysis of variance (ANOVA) or Kruskal–Wallis tests were used (as appropriate) to evaluate differences in patient satisfaction scores by year. For patients completing both the hospital and at least one ambulatory survey, Pearson’s r and Spearman correlation tests were used (as appropriate) to evaluate relationships between ambulatory and hospital PG scores. The correlation coefficient (r) was used to determine if the correlation was weak (r < +/−0.3), moderate (r > ±0.3 and <±0.6), or strong (r > ±0.6 and <±1). Statistical significance was considered at *p* < 0.05.

## 3. Results

A total of 1602 unique patients completed 2270 ambulatory surveys, and 365 unique patients completed 376 hospital surveys during our study period (Table 1). The average number of days between visit/discharge date and received date for the ambulatory survey was 10 days, and 38 days for the hospital survey. The mean age of the ambulatory survey participants was 61.86 ± 12.85 years and 59% were female. The mean age of the hospital survey participants was 65.32 ± 10.78 years, 57% were female, the mean length of stay (LOS) was 2.53 ± 1.92 days, and 83% were discharged to their own home. Thirty-two percent of patients who took the hospital survey also took one or more ambulatory surveys (Table 1).

Linear regression analyses showed that patients whose discharge disposition was “another facility” were significantly older ($\bar{x}$ = 74.0 years) compared to those discharged to “own home” ($\bar{x}$ = 64.44) or those discharged to “another home” ($\bar{x}$ = 57.82; *p* < 0.0001). Patients whose discharge disposition was “another facility” had significantly higher LOS ($\bar{x}$ = 5.07 days) compared to those discharged to “own home” ($\bar{x}$ = 2.17) or those discharged to “another home” ($\bar{x}$ = 2.88; *p* < 0.0001). There were no statistically significant correlations between other hospital PG survey questions (i.e., “Doctors Overall”, “Likelihood of Recommending”, and “Survey Overall”) and discharge disposition, age, gender, or LOS. However, in the ambulatory population, age was positively correlated with “Likelihood to Recommend” (*p* < 0.0001), “Care Provider Overall” (*p* < 0.0001), and “Survey Overall” (*p* < 0.0001).

### 3.1. Trends in PG Scores over Time

A significant change in ambulatory overall survey averages in the neurosurgeon group overall (*p* = 0.0003) and among individual surgeons (Surgeons A, B, and D) was observed over the study period. There were no other significant changes in any of the other PG scores, ambulatory or hospital, over the study period (Supplementary Materials: Tables S1 and S2).

### 3.2. Relationships within and between Ambulatory and Hospital PG Scores

**Figure 2:**
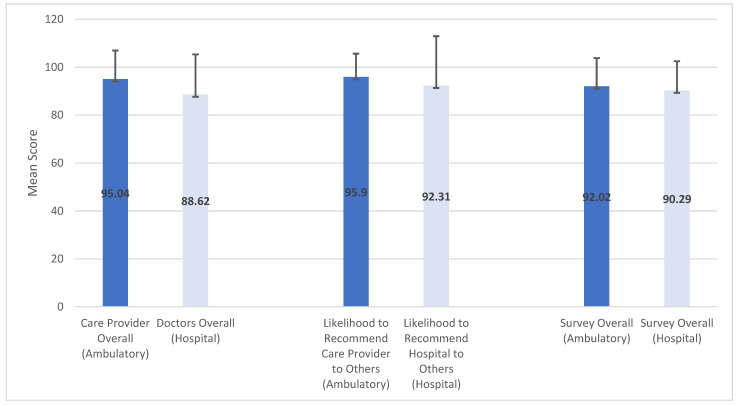
Mean and standard deviation histogram of Press Ganey scores for hospital survey patients who also completed at least one ambulatory survey.

Overall, ambulatory PG scores were higher than hospital scores. Figure 2 shows a histogram of the mean scores with standard deviation bars. Spearman correlation analyses were used to examine relationships within and between the group that completed both the hospital and ambulatory surveys (*N* = 120) and individual surgeons’ ambulatory and hospital PG scores. However, a few patients skipped or missed answering certain questions on either survey, so the sample sizes for each question ranges between 117 and 120. Within the hospital survey cohort, there were moderate to strong correlations between “Doctors Overall”, “Likelihood to Recommend Hospital”, and “Survey Overall”. This trend was also true for “Care Provider Overall”, “Likelihood to Recommend Care Provider”, and “Survey Overall” within the ambulatory survey (Table 2).

An analysis of correlations between ambulatory and hospital PG surveys found weak, yet statistically significant, negative correlations with hospital “Likelihood to Recommend” and ambulatory “Care Provider Overall” (r = −0.20421, *p* = 0.0279), ambulatory “Likelihood to Recommend” (r = −0.19622, *p* = 0.0356), and ambulatory “Survey Overall” (r = −0.28482, *p* = 0.0019). Additionally, there were weak, yet significant, negative correlations between hospital “Survey Overall” and ambulatory “Care Provider Overall” (r = −0.22351, *p* = 0.0145) and ambulatory “Survey Overall” (r = −0.21684, *p* = 0.0174). Although not statistically significant, there were consistent negative correlations between all other ambulatory and hospital PG questions (Table 2).

Examination by individual surgeon also revealed weak, negative correlations between ambulatory and hospital PG scores.

## 4. Discussion

Research has established a strong connection between the patient–provider relationship and patient satisfaction [15,16,17]. This relationship was reinforced in our analyses, where we found significant moderate to strong positive correlations between “Care Provider Overall” and “Likelihood to Recommend Care Provider” and “Survey Overall” within the ambulatory survey, and “Doctor’s Overall” and “Likelihood to Recommend Hospital” and “Survey Overall” within the hospital survey. This suggests that patient’s view of their doctor plays a critical role in patient satisfaction.

Our investigation specifically sought to understand whether, for medical providers who have direct interactions with patients in multiple settings (in the ambulatory setting and the hospital), there is a relationship between patient satisfaction scores across those different settings. Overall, we found that ambulatory scores were higher than hospital scores. Higher scores on the ambulatory surveys could be attributable to various factors, including differences in patients’ physical and mental state at the time of ambulatory visits (e.g., having less anxiety, insomnia, or pain), as compared to their physical and mental conditions post-operatively at the time of the hospital surveys. Lower scores on the hospital surveys could also be attributable, in part, to survey timing. Several studies have investigated survey timing and patient satisfaction and found that scores were poorer when measured at a longer time after the encounter [19,20,21,22,23]. Our hospital surveys were mailed, and the average time from discharge to return date was 38 days, compared to an average of 10 days between visit and return date for the ambulatory survey. This could reflect a scenario where hospital patients are more likely to actively be in the recovery phase following operative procedures, and their satisfaction scores could be strongly related to their clinical outcomes [21].

Our analysis (the first of its kind to our knowledge) also found weak, yet significant and consistent negative correlations between ambulatory and hospital PG scores. Although the correlations were weak and negative (likely due to hospital scores being lower overall than ambulatory), the significant relationships between the two could still mean that improvement initiatives in one care setting may impact scores in the other. For example, thorough pre-operative patient education and realistic short- and long-term post-operative expectation setting could improve patient satisfaction scores [24].

With so many factors going into how patients perceive and then rate their care experiences, an additional question for medical providers practicing in multiple settings was whether their patient satisfaction scores were comparable between these settings? We found that consistently, ambulatory scores were higher than hospital scores. When patients are inpatients in the hospital, they are exposed to a much higher number of interactions with various care providers over a longer time period compared to what and who they are exposed to during ambulatory clinic visits. These complex exposure differences could explain the differences in patient satisfaction scores in the different settings. As healthcare organizations, medical groups and practices are beginning to factor in patient experience scores with provider’s pay-for-performance incentives [25], it is worth thinking about the disparities between providers who only see patients in one setting (e.g., Primary Care Physicians) versus those specialists (e.g., neurosurgeons) who practice in multiple settings. Providers practicing in one setting may have more control over patient experience and therefore an advantage in pay-for-performance incentives compared to specialists practicing in multiple settings, where more confounding exposures could influence patient experience. For specialists, healthcare organizations should analyze patient experience data in the various settings to determine which patient scores best reflect provider performance and ultimately should get used to determine incentives.

This study had several limitations. Both ambulatory and hospital patient satisfaction scores can be affected by several different factors that were not specifically examined as part of this study. On the hospital side, this includes patients’ perceptions of their interactions with nurses, the hospital environment, and communication about medications, the discharge process, personal issues, and meals. On the ambulatory side, this includes patients’ perceptions on facility access, navigating the clinic, personal safety, among other factors. The patients who completed the PG hospital surveys (65.32 ± 10.78 years) were significantly older than the overall patient population (59.78 ± 14.50) of the neurosurgery practice during the same study period (*p* < 0.0001). We also noted that age was positively correlated with ambulatory PG scores, which could reflect some participation bias in the PG surveys analyzed. We also only included hospital surveys for neurosurgeons who were also listed as the attending physician, which eliminated a large number of potential hospital surveys from our analyses. Furthermore, neurosurgeons operated at various hospital facilities and saw patients in various ambulatory clinic locations, and each facility has their own individual nuances that can impact patient satisfaction scores. This was not investigated nor controlled for in our analyses. Finally, we cannot conclude that our findings represent a cause–effect relationship between ambulatory and hospital PG scores. Carefully controlled prospective studies are needed to investigate if such causative relationships exist while controlling for extraneous variables that may influence those relationships.

## 5. Conclusions

The overall case for a focus on patient experience, particularly patient satisfaction, is well established in healthcare from both business and clinical perspectives. As hospitals, medical groups, and physician practices are looking to allocate resources towards the most meaningful strategies for quality improvement, it may be worth acknowledging the potential translational power of patient perception across different care settings. The patient perception established in ambulatory and clinic settings could translate to patient’s perception of their hospital experience and subsequent satisfaction scores, and thereby represent an important focus of performance improvement initiatives. Additionally, when setting incentives based on patient satisfaction, healthcare organizations should be cognizant of the differences in patient satisfaction for providers who practice in one versus multiple settings and then carefully analyze how to fairly determine provider achievement.

## Figures and Tables

**Table 1:** Press Ganey survey participant demographics.

Demographic Variables	Values
AMBULATORY CLINIC SURVEY (*N* = 2270)	
Age, y, mean ± SD	61.86 ± 12.85
Male	793 (41.26)
HOSPITAL SURVEY (*N* = 376)	
Age, y, mean ± SD	65.32 ± 10.78
Length of Stay, d	2.53 ± 1.92
Male	161 (42.82)
Discharge Location	
Another facility	43 (11.44)
Another home	17 (4.52)
Own home	314 (83.51)
Missing	2 (0.53)
Percent of unique hospital survey patients who also completed ambulatory survey, *n* (%)	120 (32.88)

**Table 2:** Spearman correlation coefficients of hospital survey patients who also completed at least one ambulatory survey.

Spearman Correlation Coefficients Prob > |r| under H0: Rho = 0						
	Doctor’s Overall (Hospital)	Likelihood to Recommend Hospital to Others (Hospital)	Survey Overall (Hospital)	Care Provider Overall (Ambulatory)	Likelihood to Recommend Care Provider to Others (Ambulatory)	Survey Overall (Ambulatory)
Doctors Overall (Hospital)		0.43830 <0.0001	0.74920 <0.0001	−0.13166 0.1571	−0.11428 0.2219	−0.07933 0.3931
Likelihood to Recommend Hospital to Others (Hospital)	0.43830 <0.0001		0.59649 <0.0001	−0.20421 0.0279	−0.19622 0.0356	−0.28482 0.0019
Survey Overall (Hospital)	0.74920 <0.0001	0.59649 <0.0001		−0.22351 0.0145	−0.11474 0.2160	−0.21684 0.0174
Care Provider Overall (Ambulatory)	−0.13166 0.1571	−0.20421 0.0279	−0.22351 0.0145		0.69080 <0.0001	0.65218 <0.0001
Likelihood to Recommend Care Provider to Others (Ambulatory)	−0.11428 0.2219	−0.19622 0.0356	−0.11474 0.2160	0.69080 <0.0001		0.63433 <0.0001
Survey Overall (Ambulatory)	−0.07933 0.3931	−0.28482 0.0019	−0.21684 0.0174	0.65218 <0.0001	0.63433 <0.0001	

## Data Availability

The data that support the findings of this study are available from Methodist Health System but restrictions apply to the availability of these data, which were used under license for the current study, and so are not publicly available. Data are however available from the authors upon reasonable request and with permission of Methodist Health System.

## Supplementary Materials

The following are available online at https://www.mdpi.com/article/10.3390/healthcare9091153/s1, Table S1: Total neurosurgeon practice’s ambulatory and hospital mean Press Ganey scores by year, Table S2: Surgeon’s ambulatory and hospital mean Press Ganey scores by year.
