# Supplementary material for: Investigating the Relationship between Ambulatory and Hospital Patient Experience Scores in a Neurosurgery Practice
Source: Healthcare (Basel). 2021 Sep 3;9(9):1153. doi: 10.3390/healthcare9091153 (PMC8469641; doi:10.3390/healthcare9091153)
Supplement: Supplementary file 1 [file healthcare-09-01153-s001.zip › healthcare-1320297-supplementary.pdf]

Supplementary Materials

Table S1. Total neurosurgeon practice's ambulatory and hospital mean Press Ganey scores by year

PG Questions	Year										p-Value
		2016		2017		2018		2019		2020	
	N	Mean ± SD	N	Mean ± SD	N	Mean ± SD	N	Mean ± SD	N	Mean ± SD	
AMBULATORY CLINIC											
Care Provider Overall	178	95.35 ± 10.59	399	94.30 ± 13.63	506	91.25 ± 18.22	584	91.41 ± 18.88	582	93.68 ± 14.51	0.0740
Likelihood to Recommend Care Provider to Others	174	94.54 ± 12.57	392	94.20 ± 15.58	506	91.45 ± 19.77	574	91.42 ± 21.03	569	92.84 ± 17.87	0.3011
Survey Overall	179	92.13 ± 11.34	404	91.33 ± 11.38	512	89.60 ± 14.47	586	91.22 ± 13.34	586	92.62 ± 12.10	0.0003
HOSPITAL											
Doctors Overall	36	91.32 ± 11.88	100	89.71 ± 13.65	91	90.71 ± 16.77	55	90.76 ± 16.49	90	91.11 ± 14.98	0.6169
Likelihood to Recommend Hospital to Others	36	97.92 ± 7.01	100	92.75 ± 17.15	91	95.89 ± 15.86	55	92.73 ± 18.43	90	91.68 ± 20.48	0.2571
Survey Overall	37	93.62 ± 7.94	100	90.50 ± 12.31	91	92.75 ± 11.41	57	92.04 ± 12.30	91	89.61 ± 11.72	0.2582

Supplementary Materials

Table S2. Surgeon's ambulatory and hospital mean Press Ganey scores by year

**Question by Surgeon**	**Year**										***p*-Value**
	2016		2017		2018		2019		2020		
	N	Mean ± SD	N	Mean ± SD	N	Mean ± SD	N	Mean ± SD	N	Mean ± SD	
AMBULATORY CLINIC											
Care Provider Overall											
Surgeon A	18	97.92 ± 6.06	46	93.43 ± 12.64							0.1266
Surgeon B	66	94.60 ± 12.87	166	95.12 ± 12.06	188	91.87 ± 14.52	193	92.57 ± 15.51	169	93.88 ± 12.40	0.1523
Surgeon C	57	94.92 ± 9.34	65	95.29 ± 10.13	108	93.19 ± 19.44	142	90.37 ± 19.35	132	92.90 ± 15.67	0.3219
Surgeon D	30	96.04 ± 9.91	71	91.93 ± 19.07	94	91.82 ± 17.35	120	91.72 ± 19.74	105	94.10 ± 15.88	0.4091
Surgeon E			5	86.25 ± 30.75	56	84.23 ± 25.18	80	87.30 ± 26.50	62	90.91 ± 18.54	0.4768
Surgeon F	7	96.43 ± 9.45	46	95.34 ± 11.48	60	91.46 ± 19.15	49	95.82 ± 9.37	82	94.54 ± 13.51	0.6926
Surgeon G									32	97.66 ± 6.35	
Likelihood to Recommend Care Provider to Others											
Surgeon A	17	97.06 ± 8.30	45	95.00 ± 11.43							0.5528
Surgeon B	65	93.46 ± 15.48	165	95.91 ± 13.61	187	92.25 ± 16.40	191	93.19 ± 18.48	163	93.87 ± 13.63	0.0719
Surgeon C	56	95.09 ± 10.02	63	94.84 ± 12.83	109	94.50 ± 18.43	140	90.00 ± 22.60	129	92.25 ± 18.17	0.2212
Surgeon D	29	93.97 ± 12.77	70	89.64 ± 22.32	93	91.40 ± 19.33	116	91.59 ± 20.31	102	92.40 ± 21.63	0.4875
Surgeon E			4	81.25 ± 37.50	57	82.02 ± 29.03	79	87.97 ± 26.51	63	91.27 ± 19.13	0.1442
Surgeon F	7	96.43 ± 9.45	45	94.44 ± 12.93	60	92.50 ± 19.69	48	93.75 ± 16.71	81	92.59 ± 19.54	0.9754
Surgeon G									31	95.16 ± 16.35	
Survey Overall											
Surgeon A	18	95.01 ± 7.83	47	88.49 ± 13.50							**0.0438**
Surgeon B	67	91.47 ± 13.32	168	91.87 ± 10.25	191	88.18 ± 13.65	195	91.30 ± 12.94	169	92.05 ± 12.51	**0.0130**
Surgeon C	57	92.72 ± 9.04	67	93.05 ± 9.95	109	92.34 ± 14.95	142	89.67 ± 14.29	132	92.04 ± 12.50	0.4367
Surgeon D	30	91.16 ± 12.29	71	90.03 ± 13.81	94	92.20 ± 12.04	120	92.57 ± 12.25	106	94.44 ± 10.92	**0.0384**
Surgeon E			5	87.62 ± 20.32	58	84.59 ± 18.93	80	91.06 ± 15.70	63	92.04 ± 12.92	0.0651
Surgeon F	7	90.39 ± 12.43	46	92.20 ± 9.31	60	89.91 ± 13.15	49	92.32 ± 10.08	84	91.53 ± 12.66	0.8092
Surgeon G									32	96.06 ± 7.51	
HOSPITAL											
Doctors Overall											
Surgeon A	9	93.52 ± 10.02	13	92.95 ± 13.96							0.9690
Surgeon B	14	88.69 ± 10.13	28	88.39 ± 13.67	35	85.96 ± 20.26	20	94.58 ± 9.47	43	89.92 ± 16.12	0.3725
Surgeon C	8	88.02 ± 17.74	26	90.54 ± 12.11	16	90.10 ± 19.54	13	83.33 ± 26.35	14	88.69 ± 18.66	0.9644
Surgeon D	4	100.00	15	90.56 ± 10.38	15	92.22 ± 14.25	10	92.50 ± 14.41	14	95.24 ± 8.47	0.3730
Surgeon E			5	86.67 ± 19.18	17	98.04 ± 4.69	4	91.67 ± 11.79	7	86.90 ± 18.54	0.1866
Surgeon F	1	100.00	13	87.82 ± 18.51	8	94.27 ± 9.17	8	90.62 ± 13.68	10	95.00 ± 8.96	0.8708
Surgeon G									2	100.00	
Likelihood to Recommend Hospital to Others											
Surgeon A	9	97.22 ± 8.33	13	94.23 ± 14.98							0.7370
Surgeon B	14	100.00	28	91.96 ± 16.74	35	95.03 ± 20.69	21	91.67 ± 22.82	44	89.80 ± 23.61	0.1188
Surgeon C	8	93.75 ± 11.57	26	93.27 ± 16.67	16	92.19 ± 19.83	13	96.15 ± 9.39	14	89.29 ± 21.29	0.9191
Surgeon D	5	100.00	15	95.00 ± 10.35	15	96.67 ± 8.80	9	91.67 ± 25.00	14	98.21 ± 6.68	0.7696
Surgeon E			5	95.00 ± 11.18	17	98.53 ± 6.06	4	87.50 ± 14.43	6	91.67 ± 12.91	0.1653
Surgeon F			13	88.46 ± 28.17	8	100.00	8	93.75 ± 11.57	10	92.50 ± 23.72	0.4607
Surgeon G									2	100.00	
Survey Overall											
Surgeon A	9	93.48 ± 9.19	13	92.57 ± 11.41							0.8584
Surgeon B	14	93.73 ± 6.42	28	90.58 ± 11.72	35	91.62 ± 13.92	21	93.70 ± 13.43	44	89.16 ± 12.80	0.3236
Surgeon C	8	90.35 ± 10.91	26	90.85 ± 10.18	16	88.62 ± 14.56	14	87.03 ± 15.19	14	88.72 ± 12.99	0.9719
Surgeon D	5	98.19 ± 2.48	15	89.68 ± 11.45	15	92.98 ± 8.07	10	94.97 ± 6.74	14	90.65 ± 7.76	0.1712
Surgeon E			5	91.11 ± 9.99	17	97.24 ± 3.38	4	89.58 ± 10.17	7	86.45 ± 16.12	0.1125
Surgeon F	1	96.88	13	88.25 ± 19.94	8	96.04 ± 4.04	8	94.01 ± 9.17	10	93.58 ± 7.13	0.9619
Surgeon G									2	89.58 ± 7.37	
